# Lhx4 surpasses its paralog Lhx3 in promoting the differentiation of spinal V2a interneurons

**DOI:** 10.1007/s00018-024-05316-x

**Published:** 2024-07-06

**Authors:** Estelle Renaux, Charlotte Baudouin, Damien Marchese, Yoanne Clovis, Soo-Kyung Lee, Françoise Gofflot, René Rezsohazy, Frédéric Clotman

**Affiliations:** 1 Université catholique de Louvain, Louvain Institute of Biomolecular Science and Technology, Animal Molecular and Cellular Biology, Louvain-la-Neuve, 1348 Belgium; 2 Université catholique de Louvain, Institute of Neuroscience, Laboratory of Neural Differentiation, Brussels, 1200 Belgium; 3 Pediatric Neuroscience Research Program, Papé Family Pediatric Research Institute, Department of Pediatrics, Oregon Health and Science University, Portland, OR 97239 USA; 4 Department of Biological Sciences, University at Buffalo, Buffalo, NY 14260 USA

**Keywords:** Embryonic spinal cord, Paralog factors, LIM-HD transcription factors, V2 interneurons, Motor neurons, Differentiation

## Abstract

Paralog factors are considered to ensure the robustness of biological processes by providing redundant activity in cells where they are co-expressed. However, the specific contribution of each factor is frequently underestimated. In the developing spinal cord, multiple families of transcription factors successively contribute to differentiate an initially homogenous population of neural progenitors into a myriad of neuronal subsets with distinct molecular, morphological, and functional characteristics. The LIM-homeodomain transcription factors Lhx3, Lhx4, Isl1 and Isl2 promote the segregation and differentiation of spinal motor neurons and V2 interneurons. Based on their high sequence identity and their similar distribution, the Lhx3 and Lhx4 paralogs are considered to contribute similarly to these processes. However, the specific contribution of Lhx4 has never been studied. Here, we provide evidence that Lhx3 and Lhx4 are present in the same cell populations during spinal cord development. Similarly to Lhx3, Lhx4 can form multiproteic complexes with Isl1 or Isl2 and the nuclear LIM interactor NLI. Lhx4 can stimulate a V2-specific enhancer more efficiently than Lhx3 and surpasses Lhx3 in promoting the differentiation of V2a interneurons in chicken embryo electroporation experiments. Finally, *Lhx4* inactivation in mice results in alterations of differentiation of the V2a subpopulation, but not of motor neuron production, suggesting that Lhx4 plays unique roles in V2a differentiation that are not compensated by the presence of Lhx3. Thus, Lhx4 could be the major LIM-HD factor involved in V2a interneuron differentiation during spinal cord development and should be considered for in vitro differentiation of spinal neuronal populations.

**Supplementary Information:**

The online version contains supplementary material available at 10.1007/s00018-024-05316-x.

## Introduction

Paralog factors are usually described as promoting robustness in biological systems by displaying redundant functionality in the same cells [1]. The LIM-homeodomain (LIM-HD) transcription factors Lhx3 and Lhx4 have been considered to exert identical roles, and thereby to provide redundant functionalities, during the segregation of motor neurons (MNs) versus V2 interneurons (INs) and the differentiation of V2a INs in the developing spinal cord [2–4]. However, the ability of Lhx4 to individually stimulate the MN or the V2 IN differentiation programs has never been addressed.

During spinal cord development, different neuronal populations are generated from distinct progenitor domains orderly distributed along the dorsoventral axis of the ventricular zone (reviewed in [5, 6]). Lhx3 is produced early in MNs and in V2 precursors and is maintained at later stages in MNs of the medial motor column (MMC) and in V2a INs [4, 7]. At early stages, it regulates the segregation between the MN and the cardinal V2 IN lineages [7, 8], and later on promotes the differentiation of V2a INs [9]. It contributes to the segregation of MN and V2 IN lineages by forming distinct transcriptional complexes in MN and in V2 precursors. In differentiating MNs, Lhx3 associates with the LIM-HD protein Isl1 and the ubiquitous nuclear LIM interactor (NLI, also called CLIM2 or LDB-1) to form an hexameric 2Lhx3-2Isl1-2NLI complex. This complex binds hexamer-response elements (HxREs), stimulates expression of a wide range of MN genes, including *Hb9*, and promotes MN differentiation [7, 8, 10–15] while inhibiting multiple IN determinants [10]. In differentiating V2 INs, which are devoid of Isl proteins, Lhx3 associates with NLI to form a tetrameric 2Lhx3-2NLI complex that binds tetramer-response elements (TeREs) [7, 8] and stimulates *Chx10* expression, which promotes V2 IN differentiation [7–9]. Activation of irrelevant differentiation programs is mutually prevented in each cell type. In MNs, Hb9 hinders binding of tetrameric 2Lhx3-2NLI complex to TeREs [8, 9]. In V2 INs, Vsx1 and Chx10 sequentially block irrelevant activation of HxREs [9, 16].

However, as frequently observed in spinal populations [6, 17–19], these transcription factors are coexpressed along with at least one of their paralogs. Lhx3 and Lhx4 are highly conserved through evolution with respectively 94% and 98% global identity between human and mouse proteins. Lhx4 is highly similar to Lhx3, with 66% global identity, but their protein-interacting LIM domains or their DNA-binding homeodomains are even more conserved: 77% identity between the LIM1 domains, 86% identity between the LIM2 domains and 95% identity between the homeodomains, in both human and mouse [20, 21]. These paralog factors are reported to be present in the same cells in the retina, pituitary gland, hindbrain and spinal cord during embryonic development [2, 4, 20, 22–26]. As expected, they display similar activities in different cell types and contexts. In the pituitary gland, they are both necessary for the late development of the Rathke’s pouch [27] and implicated in the activation of the Follicle Stimulating Hormone β-subunit gene expression [28]. In the hindbrain, Lhx3 and Lhx4 can both associate with Isl1 to promote the expression of *Slit2* in somatic MNs [2]. During zebrafish spinal development, they act redundantly to regulate the differentiation of MNs and INs, including the late development of V2a and V2b INs [3]. In the mouse, embryos lacking either Lhx3 alone or Lhx4 alone show no defect in MN differentiation, axonal projections, or distribution. In contrast, combined inactivation of these factors results in cell fate switch from ventrally-located and projecting MNs into dorsally-located and projecting MNs in the cervical spinal cord, demonstrating functional redundancy [4]. However, the fate of MNs at other axial levels remains unknown. The LIM-HD paralog factors Isl1 and Isl2 are even more conserved through evolution with respectively 100% and 99% global identity between human and mouse proteins. Isl2 is highly similar to Isl1, with 75% global identity, but 93% identity between their LIM1 domain, 75% and 73% identity for their LIM2 domain, and 100% identity for their homeodomain, in human and mouse, respectively. The individual roles of Isl1 and Isl2 in the developing spinal cord have been investigated. In spinal MNs, they cooperate to consolidate the MN fate and prevent illegitimate activation of the V2 IN differentiation program [29–31]. Consistently, different studies suggested that Isl2 can integrate, like its paralog Isl1, in hexameric complexes able to simulate MN fate [30, 32, 33].

In contrast, in other cellular contexts, LIM-HD factors can fulfill individual functions that are not systematically shared or rescued by their paralog. Indeed, mouse Isl2-deficient embryos display defective differentiation of visceral MNs that is not observed in the absence of Isl1 [29–31], although the involved mechanism remains controversial [34]. At later stages, Isl2 individually regulates MN distribution, axonal projections, neuromuscular junction formation and sensorimotor connectivity [35]. In the retina, Lhx4 cooperates with Isl1 upstream of Lhx3 to regulate the differentiation of rod bipolar cell and selective rod-connecting bipolar cell subtypes [36]. Lhx4 is crucial for the control of *Slit2* transcription in branchiomotor neurons for it is the only Lhx paralog expressed in this population [2]. In the zebrafish, Lhx3 is sufficient to specify the identity of spinal circumferentially descending INs [3]. In the mouse, Lhx3 is sufficient in MNs to impose the acquisition of a medial motor column phenotype [37]. However, whether the murine Lhx3 and Lhx4 paralogs exert redundant or individual and specific roles during MN and V2 IN segregation and differentiation remains unknown. Differences in the binding of Lhx3 and Lhx4 proteins to Isl1 and Isl2 have been reported and could indicate a divergence in function [32]. Interestingly, binding measurements show that the Isl2-Lhx4 complex displays an eightfold higher *Kd* than the Isl1-Lhx3 equivalent, suggesting that Isl2-Lhx4 could be more effective than the canonical Isl1-Lhx3 pair for MN differentiation [33].

Taken together, these observations raise the question of the importance of Lhx4 for spinal MN and V2 IN segregation and differentiation. Here, we show that Lhx4 can, similarly to Lhx3, form complexes with Isl1 or Isl2 and NLI. Using cultured cells and chicken embryonic spinal cord electroporation, we determine that Lhx4 and Isl2 are as efficient as Isl1 and Lhx3 to stimulate the activity of a MN HxRE and to promote the differentiation of spinal MNs. In contrast, Lhx4 is more efficient than Lhx3 to stimulate the activity of a V2 TeRE and to promote the differentiation of spinal V2a INs. Furthermore, we analyzed the phenotype of constitutive or conditional *Lhx4*-deficient mouse embryos. While the loss of Lhx4 in MNs seems compensated by the presence of Lhx3, the loss Lhx4 in V2 INs results in a reduction in the differentiation of V2a INs. Taken together, these results suggest that Lhx4 could be the major LIM-HD paralog involved in V2a differentiation during spinal cord development and should be considered for in vitro differentiation protocols of V2a INs.

## Materials and methods

### Mouse lines

*Olig2-Cre*, *Vsx1-CreER*^*T2*^, *PGK-Cre*, and *Lhx4*^*loxP/loxP*^ (kindly provided by Dr L. Gan) mice have been previously described [38–41]. The *Lhx4*^*+/loxP*^ line was crossed with *PGK-Cre* line to obtain *Lhx4*^*+/-*^ mice. *Lhx4*^*+/-*^ mice were then crossed to generate *Lhx4*^*-/-*^ constitutive knockout embryos, using *Lhx4*^*+/+*^ embryos as controls. The *Lhx4*^*loxP/loxP*^ line was crossed with *Olig2-Cre* or *Vsx1-CreER*^*T2*^ transgenic mice bearing heterozygous-null mutations for Lhx4 (*Olig2-Cre|Lhx4*^*+/-*^ or *Vsx1-CreER*^*T2*^*|Lhx4*^*+/-*^) to obtain *Vsx1|Lhx4*^*Δ/-*^ or *Olig2|Lhx4*^*Δ/-*^ conditional knockout embryos. Control embryos for those two conditional knockouts were *Vsx1|Lhx4*^*+/-*^ and *Olig2|Lhx4*^*+/-*^, respectively. The morning of the vaginal plug was considered as embryonic day (E)0.5. The embryos were collected at embryonic day (E)14.5, and 5 embryos for each genotype were analyzed in each experiment. For crossings with *Vsx1-CreER*^*T2*^ transgenic mice, pregnant females were injected intraperitoneally with tamoxifen (100 mg/kg) twice at E.9.5 to activate inducible CreER^T2^ activity [40].

### Plasmids

Reporter or expression plasmids used were: TeRE::GFP, HxRE::GFP, TeRE::Luc, HxRE::Luc, pCS2-Myc-Isl1, pCS2-HA-Lhx3 and pCS2-Isl1::Lhx3 [8, 10], and empty-pCS2, pCS2-HA-Lhx4 wherein the coding sequence of Lhx3 was replaced with that of Lhx4, pCS2-Myc-Isl2 wherein the coding sequence of Isl1 was replaced with that of Isl2, and pCS2-Isl1::Lhx4, pCS2-Isl2::Lhx3 and pCS2-Isl2::Lhx4 wherein the coding sequence of Lhx3 or Isl1 was replaced with that of Lhx4 or Isl2, preserving the flexible arm between the two parts of the fusion protein [10] (details about plasmid generation available upon request). Electroporation control vectors were pCAGGS::DsRed2 (kindly provided by Y. Takahashi [42]) or pCMV-GFP (kindly provided by C.Pierreux).

### Cell culture

Embryonic carcinoma P19 cells were maintained in culture flasks at 37 °C under 5% CO_2_. D-MEM medium (Gibco, #61965-059) was supplemented with 10% fetal bovine serum (Fisher scientific #10270–106) and 100 U/ml penicillin-streptomycin (Thermofisher #15140122). The cells were subcultured when they reached 80% confluency.

### Co-immunoprecipitation

P19 cells were seeded in 6-well plates (3.5 × 10^5^ cells per well) and transfected after 24 h using JetPrime transfection reagent (Westburg, PO 101000001) with 500ng of pCS2-HA-Lhx3 or pCS2-HA-Lhx4, pCS2-Myc-Isl1 and pCS2-Myc-Isl2, respectively. Fourty-eight hours after transfection, cells were rinsed with phosphate-buffered saline (PBS) and lysed in lysis buffer (Tris–HCl 20 mM pH 7.5, 120 mM NaCl, 0.5% NP40, 10% glycerol and protease inhibitors (#11873580001, Roche)). Cell lysates were cleared by centrifugation for 5 minutes at 5000 g and the cleared lysates were incubated for 4 h with protein G Agarose beads (Sigma # 11719416001) at 4 °C. The samples were centrifuged at 2000 g for 5 minutes. Supernatants were incubated with anti-HA antibody (Roche #12CA5) overnight at 4 °C, then incubated for 4 h with protein G-Agarose beads at 4 °C. After incubation, beads were washed 3 times with lysis buffer and processed for immunoblotting detection.

### Immunoblotting

Samples were loaded onto an 8% acrylamide gel, then transferred to a nitrocellulose membrane. The membrane was blocked for 1 h at room temperature in a blocking solution consisting of TBS 1x, 0.1% Tween-20 (TBS-T) and 5% powdered milk. The membrane was then incubated overnight at 4 °C with the primary antibodies: mouse anti-CLIM1/2 (Santa Cruz Biotechnology #sc-376030) at 1:1000, rabbit anti-HA-Tag (Cell Signaling #C29F4) at 1:2000, or rabbit anti-Myc-Tag (Cell Signaling #71D10) at 1:5000 in a TBS 1x, 0.1% Tween-20 (TBS-T) and 1% powdered milk solution. The membrane was washed three times in TBS-T for 10 min at room temperature, then incubated for 1 h with rabbit HRP-conjugated secondary antibody against the primary antibody (Santa cruz sc-2357) diluted in blocking buffer (1:20.000). The membrane was finally revealed using ECL chemiluminescence (Perkin Elmer NEL105001EA).

### Luciferase assay

Luciferase assays were performed using the Dual-luciferase Reporter Assay System (Promega #E1910). P19 cells were seeded in 24-wells plates (1.75 × 10^5^ cells per well) and transfected after 24 h using JetPrime transfection reagent (Westburg, PO 101000001) with 125ng of target luciferase reporter plasmid (TeRE::Luc or HxRE::Luc [8]), 125ng of pCAG, pCS2-Myc-Isl1, pCS2-Myc-Isl2, pCS2-HA-Lhx3, pCS2-HA-Lhx4, pCS2-Isl1::Lhx3, pCS2-Isl1::Lhx4, pCS2-Isl2::Lhx3 or pCS2-Isl2::Lhx4 expression plasmids and 3ng of Renilla luciferase control vector (used for reporter activity normalization), respectively. After 48 h of treatment, cells were collected and prepared according to manufacturer’s instructions. Luciferase reporter activities were measured with a tube luminometer (Promega Glomax 20/20). The relative luciferase activity was determined as the ratio between the target and constitutive luciferase activities.

### In ovo electroporation

In ovo electroporation was performed at Hamburger-Hamilton (HH) stage HH15-16 and embryos were collected at HH20 (24 h after electroporation) or HH26 (72 h after electroporation). Electroporated plasmids were TeRE::GFP or HxRE::GFP (2,5 µg/µl), and/or empty-pCS2, pCS2-Myc-Isl1, pCS2-Myc-Isl2 pCS2-HA-Lhx3, pCS2-HA-Lhx4, pCS2-Isl1::Lhx3, pCS2-Isl1::Lhx4, pCS2-Isl2::Lhx3 or pCS2-Isl2::Lhx4 plasmids (1 µg/µl). These vectors were co-electroporated along with the pCAGGS::DsRed2 plasmid or pCMV-GFP (0,25 µg/µl) to visualize electroporated cells.

### Immunofluorescence labeling

Collected embryos were fixed in ice-cold PBS/4% paraformaldehyde (PFA) for 15 to 25 min, depending on the developmental stage. After washes in PBS, the fixed embryos were incubated in PBS/30% sucrose overnight at 4 °C, embedded and frozen in PBS/7.5% gelatin/15% sucrose. Immunolabeling was performed on 14 μm serial cryosections as previously described [43]. Primary antibodies against the following proteins were used: sheep anti-Chx10 (Exalpha Biologicals #X1179P) at 1:200, anti-Cleaved Caspase-3 (Cell signaling #ASP 175) at 1:100, goat anti-Foxp1 (R&D #AF4534) at 1:500, rat anti-Gata3 (Absea Biotechnology #111214D02) at 1:15 or rabbit anti-Gata3 (Cell signaling #5852) at 1:200, chicken anti-GFP (Aves lab #GFP-1020) at 1:2000, mouse anti-Hb9/Mnr2 (DSHB #81.5C10) at 1:2000, goat anti-Isl1/2 (Neuromics #GT15051) at 1:1000 or mouse anti-Isl1/2 (DSHB #39.4D5) at 1:6000 or rabbit anti-Isl1 (Abcam #ab26122) at 1:1000, guinea pig anti-Lhx3 [44] at 1:250, rabbit anti-Lhx4 (Proteintech #11183-1-AP) at 1:300, guinea pig anti-MafA [45] at 1:500, rabbit anti-MafA (Novus Biological #NB400-137) at 1:500, guinea pig anti-OC1/HNF6 [43] at 1:6000, sheep anti-OC1/HNF6 (R&D # AF6277) at 1:250, rat anti-OC2 [46], rabbit anti-Olig2 (Millipore #AB9610) at 1:4000, mouse anti-Shox2 (Abcam #ab55740) at 1:200 and guinea-pig anti-Sox14 [9] at 1:1000. Following secondary antibodies were used: donkey anti-goat/AlexaFluor 594, donkey anti-mouse/AlexaFluor 594, goat anti-mouse IgG1/AlexaFluor 594, donkey anti-rabbit/AlexaFluor 594, donkey anti-rat/AlexaFluor 594, donkey anti-guinea pig/AlexaFluor594, donkey anti-rabbit/AlexaFluor 647, donkey anti-rat/AlexaFluor647, donkey anti-rabbit/AlexaFluor488, donkey anti-goat/AlexaFluor488, donkey anti-rat/AlexaFluor488, donkey anti-mouse/AlexaFluor488, donkey anti-chicken/AlexaFluor 488 and donkey anti-guinea pig/AlexaFluor488 purchased from ThermoFisher Scientific or Jackson Laboratories and used at dilution 1:500.

### Imaging

Immunofluorescence images of cryosections were acquired on an epifluorescence microscope EVOS FL Auto Imaging System (ThermoFisher Scientific), on a Zeiss AXIO Observer Z1 Inverted LED Fluorescence Motorized Microscope using ZEN Blue Zeiss software and on a confocal laser Scanning biological microscope FV1000 Fluoview with the FV10-ASW 01.02 software (Olympus) or on a confocal microscope Leica Stellaris 8 Falcon using Las X software. The images were treated with Fiji-ImageJ, Adobe Photoshop CC or Las X office softwares to match the brightness and contrast with the observations.

### Experimental design and statistical analyses

For quantifications of in ovo electroporation experiments, labeled cells were counted on 5 to 16 sections on each side of the spinal cord in 3 to 7 independent embryos per condition using the count analysis tool of Adobe Photoshop CC software or the count analysis tool of Fiji-ImageJ software. Quantifications on the “electroporated” (targeted) side of the spinal cord were compared to quantifications on the “non-electroporated” (contralateral) side of the same embryo. For quantifications in mouse embryonic models, the different levels of the spinal cord were determined using immunolabeling for Foxp1, which enables visualization of the lateral motor columns in brachial or lumbar regions. Labeled cells were counted on both sides of the spinal cord on 3 to 14 sections at brachial level, 8 to 18 sections at thoracic level and 4 to 13 sections at lumbar level for each of the 5 embryos analyzed per genotype. Quantifications in the different Lhx4 mutant embryos were compared to quantifications of control embryos from the same litters. Statistical analyses and graphs were performed using JMP Pro 17 Software. For mouse embryo analyses, differences in cell numbers between two different groups were evaluated using either a Wilcoxon-Mann-Whitney’s non parametrical test, a Welch’s *t*-test or a Student’s *t*-test, depending on the normality and the homoscedasticity of the data. Differences in experimental data among multiple groups were evaluated with either a Kruskal-Wallis’s non parametrical test followed by a Dunn’s test, a Games-Howell’s test, a Dunnett’s test or a Tuckey’s test depending on the normality and the homoscedasticity of the data to compare all the conditions to the control group or the different conditions one to another. In all statistical analyses, a value of *p* < 0.05 was defined as significant.

## Results

### Lhx4 is expressed in V2a INs and MNs in chicken and mouse embryonic spinal cord

**Fig. 1:**
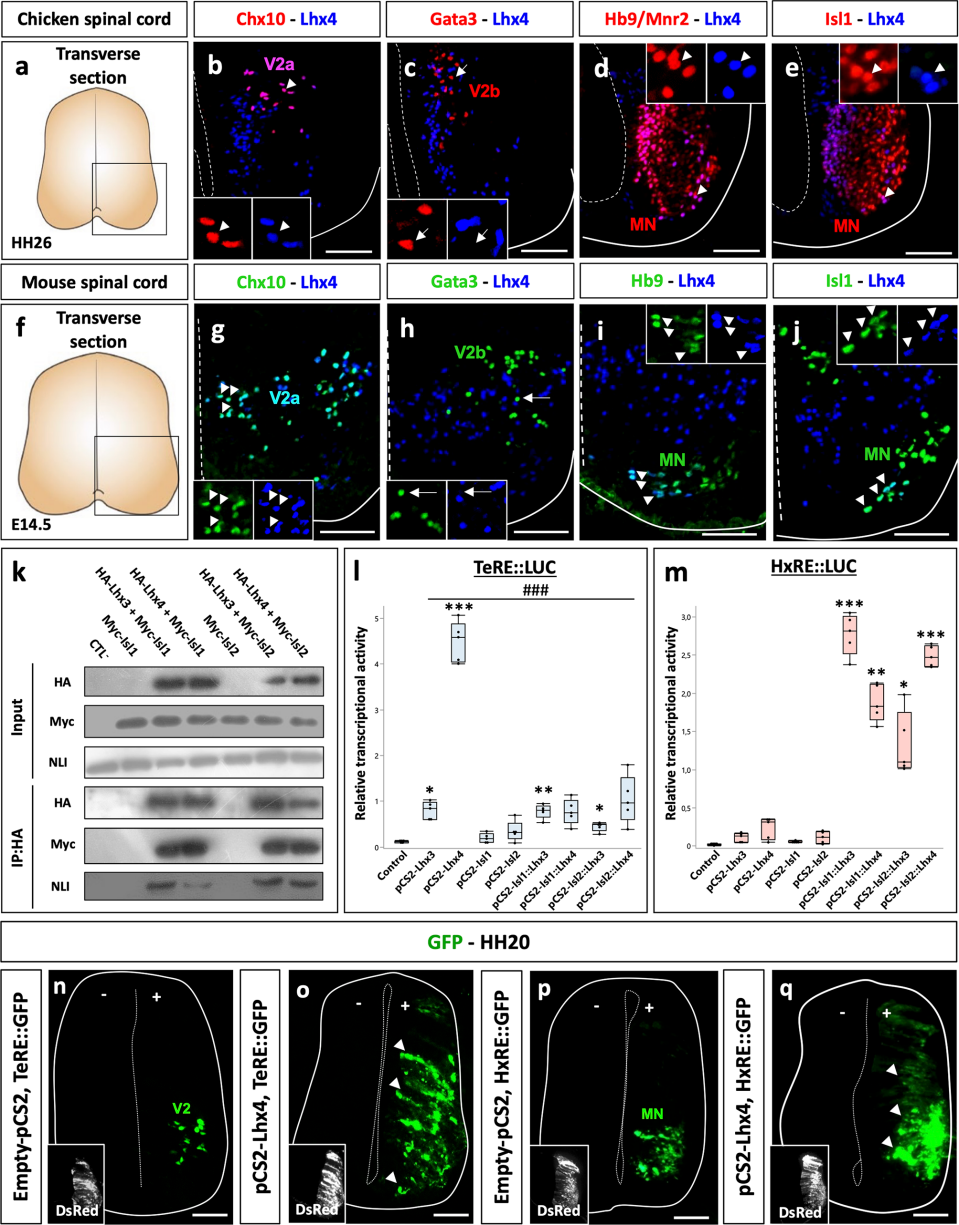
Lhx4 is produced in V2a INs and in MNs in embryonic spinal cord, can bind Isl1/2 and NLI and can stimulate TeRE and HxRE activity. **a-j** Immunofluorescence confocal images of chicken or mouse transverse embryonic spinal cord sections at stage HH26 or E14.5, respectively. Lhx4 is detected in V2a INs (Chx10+), in newly born MNs (Hb9/Mnr2+) and in differentiating MNs (Isl1+) but not in V2b INs (Gata3+). Arrowheads indicate co-detection with Lhx4. Arrows indicate absence of co-detection with Lhx4. **k** Co-immunoprecipitation experiments with HA-tagged Lhx3 or Lhx4 protein and Myc-tagged Isl1 or Isl2 in P19 cells. Input shows similar production of NLI, HA-tagged and Myc-tagged proteins in the different experimental conditions. Isl1 and Isl2 are immunoprecipitated in the presence of Lhx3 and of Lhx4, whereas endogenous NLI is immunoprecipitated in the presence of Lhx proteins with or without Isl1 or Isl2, but not with Isl1 or Isl2 alone nor with an empty vector (control). **l** Activation of the TeRE enhancer by LIM-HD factors assayed by transient transfection experiments in P19 cells. Lhx3, Lhx4 and Isl1::Lhx3 or Isl2::Lhx3 fusions proteins, which closely mimic the activity of the MN-hexameric complex by recruiting endogenous NLI, are able to activate the TeRE activity. However, Lhx4 alone is significantly more efficient in activating this V2 enhancer than Lhx3 or the Isl::Lhx3 fusions proteins. **m** Activation of the HxRE enhancer by LIM-HD factors assayed by transient transfection experiments in P19 cells. Isl or Lhx proteins alone are unable to activate the HxRE. In contrast, all the Isl::Lhx fusion proteins strongly activate this enhancer, with similar efficacy. **n-q** Lhx4 stimulates the activity of the TeRE and of the HxRE enhancers in the chicken embryonic spinal cord. TeRE::GFP or HxRE::GFP reporters were electroporated with or without an expression vector for Lhx4 at stage HH15, and embryos were collected at HH20. Co-electroporation with pCAG-dsRed2 is shown as an electroporation control. In the absence of Lhx4, TeRE activity is restricted to the V2 INs, whereas Lhx4 stimulates TeRE activity along the dorso-ventral axis of the spinal cord (arrowheads). Similarly, HxRE activity without Lhx4 is detected in MNs, but Lhx4 induces a mild activation along the dorso-ventral axis of the spinal cord (arrowheads). *n* ≥ 3. + = electroporated side; - = control side; * = *p* < 0.05; ** = *p* < 0.01; *** or ### = *p* < 0.001. Scale bars = 50 μm

Lhx4 is produced in MNs during mouse embryonic spinal cord development [4] but whether it is also expressed in V2a INs, like Lhx3, was never formally assessed, although its presence in those cells is supported by scRNAseq data [47]. Therefore, we assessed the distribution of Lhx4 in the mouse developing spinal cord using immunofluorescence. We also studied Lhx4 distribution in chicken embryonic spinal cord to determine if its expression is conserved between species and to validate the use of the chicken embryo model to assess Lhx4 activity (Fig. 1). As reported for Lhx3 [4, 7, 47], Lhx4 was similarly detected in mouse or chicken embryonic spinal cord in V2a but not in V2b INs (Fig. 1a-c, f-h), and was present in newly-born MNs and in a subset of ventral differentiating MNs (Fig. 1d-e, i-j). In mouse, both Lhx3 and Lhx4 were detected in V2a subpopulations containing the Onecut (OC) factors OC1 and OC2 (Figure S1a-d) [48, 49], in similar proportions (OC1 detected in 7.5% and 6.7% of V2a containing Lhx3 or Lhx4, respectively; OC2 present in 7.2% and 6.9% of V2a containing Lhx3 or Lhx4, respectively). In contrast, Lhx paralogs were not detected in V2a subpopulations producing MafA (Figure S1e-f) [48, 49], but were both present along with MafA in the MNs of the MMC (arrows in Figure S1e-f) [4]. Thus, the distribution of Lhx4 in MNs and V2 INs is conserved between mouse and chicken embryos and is similar to the one of Lhx3 in MMC MNs and in V2a subpopulations.

### Lhx4 can bind Isl1, Isl2 and NLI, and stimulate TeRE and HxRE

Previous studies have shown that Lhx3 stimulates V2 differentiation by interacting with NLI and forming the V2 tetramer [7–9], and promotes MN production by associating with NLI and Isl1 and forming the MN hexamer [7, 8, 10–14]. To assess if Lhx4 could contribute to V2 IN and MN differentiation using similar molecular mechanisms, we first established whether Lhx4 can associate with Isl1 or NLI. In addition, we assessed whether Isl2 could participate, like its paralog Isl1, in the formation of these complexes. Co-immunoprecipitation experiments of HA-tagged versions of Lhx3 or Lhx4 in P19 cells demonstrated that both proteins can associate with Isl1, Isl2 and endogenous NLI (Fig. 1k), suggesting that they can both participate in the formation of V2-tetrameric or MN-hexameric complexes [2, 36] and that Isl2 can also integrate into MN hexamers [30, 32, 33]. Using identical DNA concentrations for transfection, the different proteins were produced at comparable levels (see Input in Fig. 1k).

Second, to determine if Lhx4 can assemble functional complexes, we assessed whether Lhx4 can stimulate TeRE or HxRE enhancers in P19 cells in comparison to Lhx3 (Fig. 1l-m). Lhx3 alone mildly stimulated TeRE (Fig. 1l; *p* = 0.0111), as previously reported [8]. Similarly, fusion proteins between Lhx3 and Isl1 (Isl1::Lhx3) or Isl2 (Isl2::Lhx3), which closely mimic the activity of the MN-hexameric complex by recruiting NLI [10], could also slightly stimulate the TeRE (Fig. 1l; *p* = 0.005 and 0.013, respectively) [8]. In contrast, Lhx4 strongly activated this V2-enhancer (Fig. 1l; *p* = 0.0002) and did so significantly more efficiently than Lhx3 (Fig. 1l; Lhx3 *p* < 0.0001, Isl1::Lhx3 *p* = 0.0001 or Isl2::Lhx3 *p* = 0.0002), suggesting that Lhx4 may be more potent than its paralog for stimulating V2 differentiation. As expected, Isl1 or Isl2 alone, or fusion proteins between Lhx4 and Isl1 or Isl2 did not alter TeRE activity (Fig. 1l; *p* = 0.7595, 0.4746, 0.0511 and 0.1324, respectively). As for the MN enhancer, neither Lhx3, Lhx4, Isl1 nor Isl2 alone was able to activate the HxRE (Fig. 1m; *p* = 1.0000, 0.4573, 1.0000 and 1.0000, respectively), consistent with previous reports [8]. In contrast, the Isl1::Lhx3 fusion protein strongly stimulated HxRE activity (Fig. 1m; *p* < 0.0001), as described [8, 10]. A similar induction was observed with either Isl1::Lhx4, Isl2::Lhx3 or Isl2::Lhx4 fusion proteins (Fig. 1m; *p* = 0.0042, 0.0209 and 0.0002, respectively), suggesting that Lhx4 and Isl2 are at least as potent as Lhx3 and Isl1 for activating HxRE. Of note, endogenous expression of *Isl1*, *Isl2*, *Lhx3* and *Lhx4* in P19 cells remained undetectable under all the tested conditions (data not shown), suggesting that the observed effects resulted from intrinsic activity of the transfected constructs. Thus, Lhx4 alone could stimulate the V2 TeRE more efficiently than Lhx3 and, when combined with Isl1 or Isl2 in the MN hexameric complex, could promote HxRE activation as efficiently as Lhx3.

Third, to address the relevance of these observations regarding spinal neuronal differentiation, reporter constructs for the TeRE or the HxRE were co-electroporated into chicken embryonic spinal cords with an *Lhx4* expression vector at the time of MN and V2 IN differentiation. As compared to electroporation with an empty vector (Fig. 1d), Lhx4 alone ectopically stimulated the TeRE-GFP reporter construct dorsal to the V2 domain (Fig. 1o), as previously reported for Lhx3 [31] and mildly activated the HxRE-GFP reporter dorsal to the endogenous activation domain in MNs (Fig. 1p-q).

Thus, as previously reported for its paralog Lhx3, Lhx4 can integrate into V2-tetrameric or MN-hexameric complexes that are able to respectively stimulate the TeRE and HxRE enhancers in the developing spinal cord. Our data further suggest that Lhx4 could be more potent than Lhx3 in stimulating V2 enhancer activity and thereby in promoting V2 differentiation.

### Lhx4 is more efficient than Lhx3 in promoting V2a IN differentiation

**Fig. 2:**
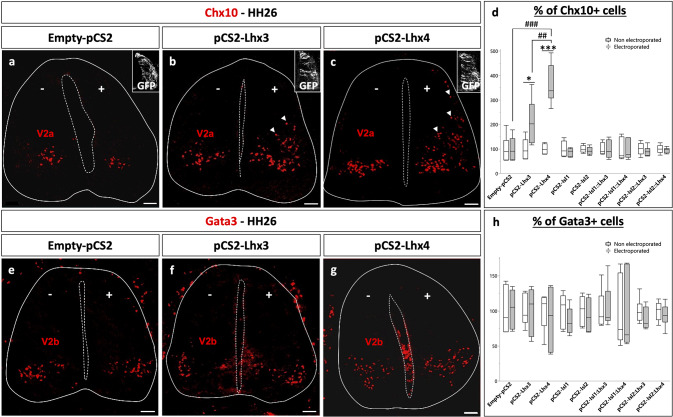
Lhx4 can stimulate the differentiation of V2a INs more efficiently than Lhx3. Immunolabeling for V2a (Chx10+) or V2b INs (Gata3+) on transverse spinal cord sections of chicken embryos electroporated with an empty vector or expression vectors for Lhx or Isl factors or the corresponding Isl::Lhx fusion proteins at stage HH15 and collected at stage HH26. Co-electroporation with pCMV-GFP is shown as an electroporation control. **a-d** As compared to an empty vector, Lhx3 and Lhx4 induce ectopic differentiation of V2a INs (Chx10+) (arrowheads) whereas Isl1, Isl2 or the different Isl::Lhx fusion proteins do not. Lhx4 is more efficient than Lhx3 in promoting V2a differentiation. **e-h** In contrast, Lhx and Isl factors or Isl::Lhx fusion proteins have no impact on V2b IN production (Gata3+). *n* ≥ 5. + = electroporated side; - = control side; * or # = *p* < 0.05; ## = *p* < 0.01; *** or ### = *p* < 0.001. Scale bars = 50 μm. See also Figure S2.1 and S2.2

Activation of the HxRE enhancer stimulates the expression of the *Hb9* MN determinant and promotes MN differentiation [8]. Similarly, TeRE stimulation promotes *Chx10* expression and V2a IN differentiation [8, 9]. To determine if Lhx4 is sufficient to induce these two processes and to compare Lhx4 with Lhx3, we first assessed the ability of these two paralogs to stimulate V2 IN differentiation. Ectopic expression of *Lhx3* along the dorsoventral axis of the spinal cord mildly stimulated differentiation of V2a INs characterized by the presence of Chx10 (Fig. 2a-b, d, Figure S2.2a-b, j; *p* = 0.037) and of Shox2 (Figure S2.1a, c; *p* = 0.0002), as previously reported [7, 8]. However, Lhx4 was also able to induce ectopic V2a IN production (Fig. 2a, c-d, Figure S2.1a, c-i; *p* = 7.3.10^− 5^; Figure S2.2b-c; *p* = 0.0003), and was more efficient than Lhx3 in stimulating V2a differentiation (Chx10: *p* = 0.0058; Shox2: *p* = 0.0281), confirming that Lhx4 surpasses its paralog in this process. In contrast, neither Isl1 or Isl2 alone, nor Isl::Lhx fusion proteins, were able to stimulate V2a differentiation (Fig. 2d, Figure S2.2 d-j; *p* = 0.433, 0.460, 0.928, 0.982, 0.475, 0.560, respectively).

Lhx3 has been shown to regulate the segregation between the MN and V2 IN lineages at early stages [7, 8], and to promote the differentiation of V2a INs at later stages [9]. To determine whether Lhx4 promotes general differentiation of V2 INs or specifically stimulates V2a production, we analyzed the distribution of the V2b-specific marker Gata3. However, none of the Lhx or Isl factors alone, or Isl::Lhx fusions proteins, had an impact on the production of V2b INs (Fig. 2e-h, Figure S2.2k-t; *p* = 0.951, 0.626, 0.333, 0.692, 0.655, 0.917, 0.266, 0.480, in order of appearance on the graph). Taken together, these observations demonstrate that Lhx4 can specifically stimulate V2a IN production and suggest that it surpasses Lhx3 in this process.

**Fig. 3:**
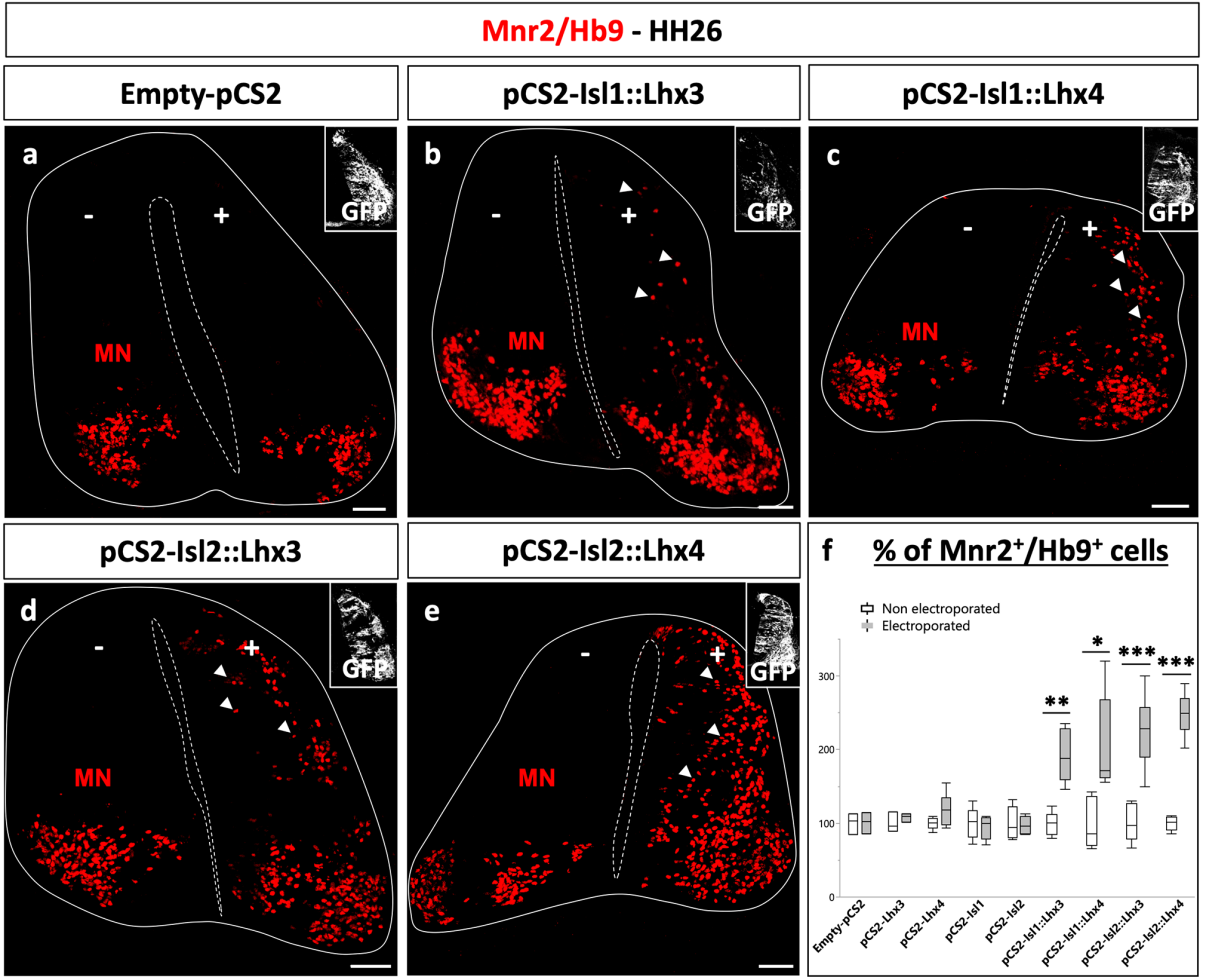
In complexes with Isl1 or Isl2, Lhx4 can induce the differentiation of MNs as efficiently as Lhx3. Immunolabeling for MNs (Mnr2/Hb9+) on transverse spinal cord sections of chicken embryos electroporated with an empty vector or expression vectors for Lhx or Isl factors or the corresponding Isl::Lhx fusion proteins at stage HH15 and collected at stage HH26. Co-electroporation with pCMV-GFP is shown as an electroporation control. **a-f** As compared to an empty vector, all the Isl::Lhx fusion proteins can stimulate MN differentiation, as evidenced by the ectopic distribution of the newly-born MN marker Mnr2, whereas the Isl or Lhx factors alone do not. *n* ≥ 3. + = electroporated side; - = control side; * = *p* < 0.05; ** = *p* < 0.01; *** = *p* < 0.001. Scale bars = 50 μm. See also Figure S3

Similarly, whereas neither Lhx3, Lhx4, Isl1 nor Isl2 alone was able to alter MN differentiation (Fig. 3f, Figure S3a-e, j; *p* = 0.416, 0.082, 0.663, 0.815, respectively), Isl1::Lhx3, Isl1::Lhx4, Isl2::Lhx3 and Isl2::Lhx4 fusion proteins induced ectopic differentiation of MNs along the dorsoventral axis of the spinal cord (Fig. 3a-f; *p* = 0.001, *p* = 0.014, *p* = 5.387.10^− 5^ and *p* = 3.788.10^− 7^, respectively). All the fusion proteins demonstrated similar efficacy regarding MN production. Thus, Lhx4 and Isl2 are as efficient as Lhx3 and Isl1 in promoting the production of MNs, but Lhx4 seems more efficient in stimulating V2a differentiation than Lhx3.

### Lhx4 is necessary for a proper differentiation of V2a interneurons

**Fig. 4:**
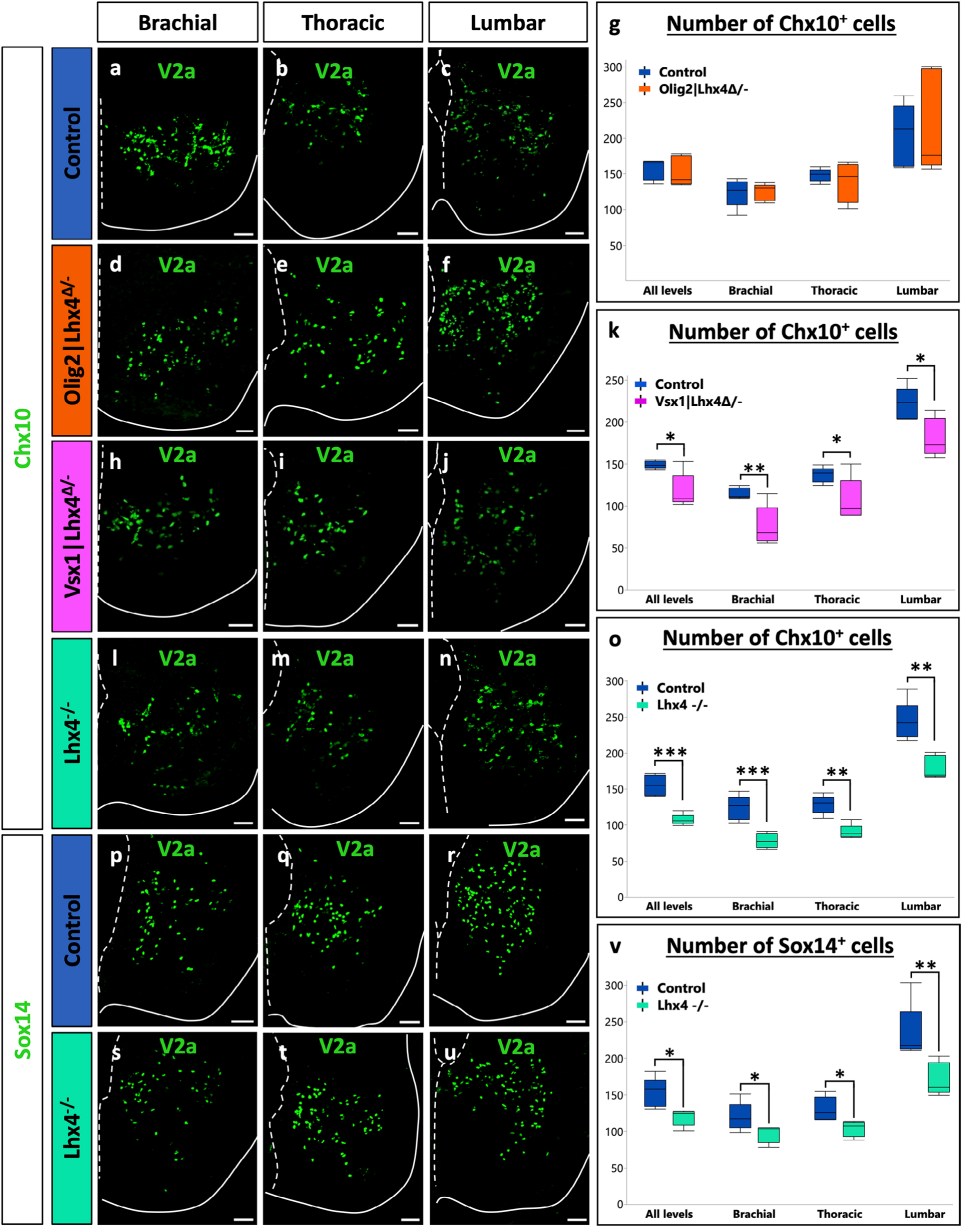
Lhx4 is necessary for proper differentiation of V2a interneurons. Immunolabeling of transverse spinal cord sections of Lhx4^+/+^ control, MN-conditional Olig2|Lhx4^Δ/−^ mutant, V2-conditional Vsx1|Lhx4^Δ/−^ mutant or Lhx4^−/−^ constitutive mutant embryos at E14.5 at brachial, thoracic, or lumbar levels. **a-g** The conditional loss of Lhx4 in MNs does not affect the production of V2a INs (Chx10+). **h-k** However, as compared to controls, embryos lacking Lhx4 only in V2 INs show a reduction in the number of Chx10 + V2a INs at all the levels of the spinal cord. **l-v** Similarly, as compared to controls, embryos constitutively lacking Lhx4 show a reduction in the number of Chx10 + or Sox14 + V2a INs at all the levels of the spinal cord. *n* = 5. * = *p* < 0.05; ** = *p* < 0.01; *** = *p* < 0.001. Scale bars = 50 μm

**Fig. 5:**
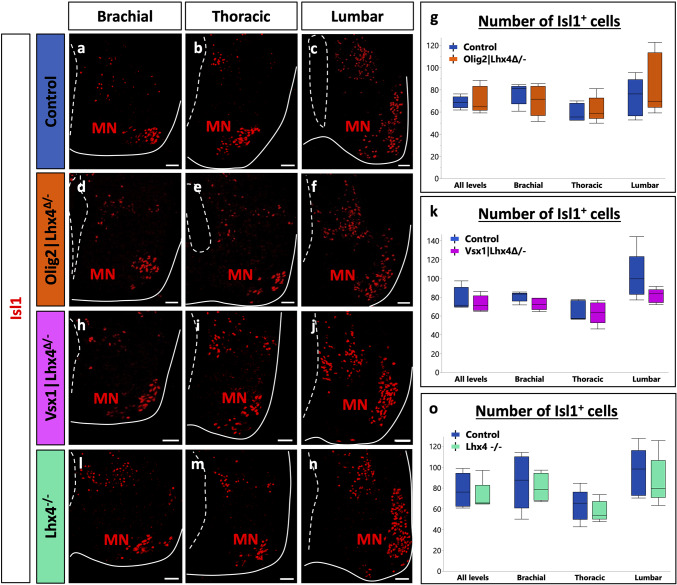
Lhx4 is not necessary for proper production of motor neurons. Immunolabeling of transverse spinal cord sections of Lhx4^+/+^ control, MN-conditional Olig2|Lhx4^Δ/−^ mutant, V2-conditional Vsx1|Lhx4^Δ/−^ mutant or Lhx4^−/−^ constitutive mutant embryos at E14.5 at brachial, thoracic, or lumbar levels. **a-o** The loss of Lhx4, whether in MNs only, in V2 INs only or in both populations, does not affect the proper production of MNs, laterally and ventrally located and characterized by the ventral expression of Isl1, as compared to controls. *n* = 5. Scale bars = 50 μm

**Fig. 6:**
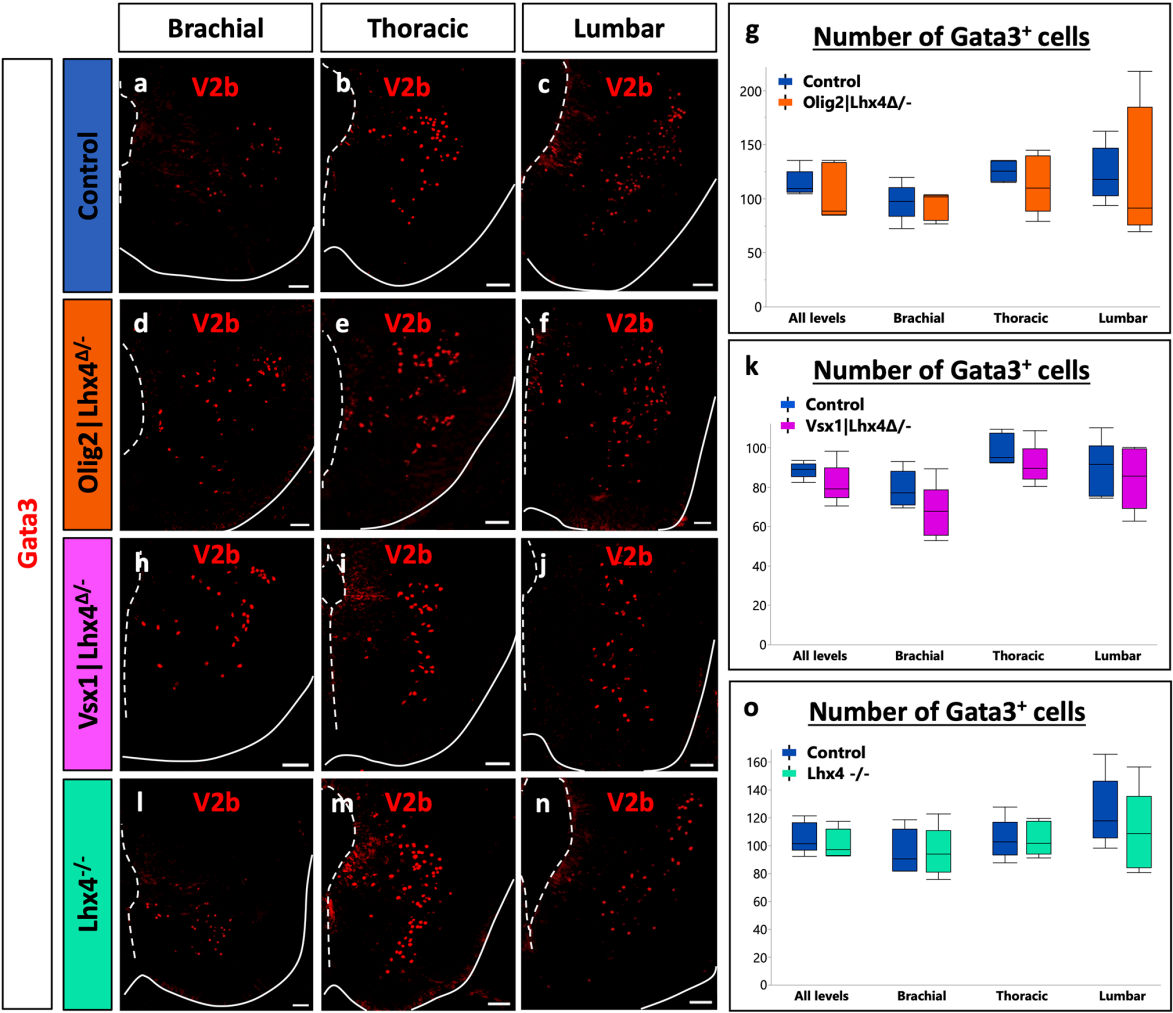
Lhx4 is not necessary for proper production of V2b interneurons. Immunolabeling of transverse spinal cord sections of Lhx4^+/+^ control, MN-conditional Olig2|Lhx4^Δ/−^ mutant, V2-conditional Vsx1|Lhx4^Δ/−^ mutant or Lhx4^−/−^ constitutive mutant embryos at E14.5 at brachial, thoracic, or lumbar levels. **a-o** The loss of Lhx4, whether in MNs only, in V2 INs only or in both populations, does not affect the proper production of Gata3 + V2b INs, as compared to controls. *n* = 5. Scale bars = 50 μm

As our previous results supported that Lhx4 can stimulate MN and V2a IN differentiation and could be even more efficient than Lhx3 for the latter, we wanted to assess whether Lhx4 is necessary for proper differentiation of these two populations or if Lhx3 can compensate for its loss. Therefore, we analyzed the phenotype of constitutive mouse mutant embryos for Lhx4 (*Lhx4*^*−/−*^) and of conditional mutant embryos where Lhx4 is absent either from the V2 INs (*Vsx1|Lhx4*^*Δ/−*^) [40] or from the MNs (*Olig2|Lhx4*^*Δ/−*^) [38]. The conditional loss of Lhx4 in V2 INs led to a significant decrease in the number of V2a INs at each level of the spinal cord (Fig. 4h-k; *p* = 0.010). Similarly, the constitutive loss of Lhx4 resulted in a reduction in the number of V2a INs (Fig. 4l-o; *p* = 0.0002). This reduction was not associated with an increased apoptosis as only a minor proportion of Chx10-positive cells contained cleaved Caspase-3 (0.36% in controls versus 0.27% in Lhx4 mutants; data not shown). This reduction corresponded to a decrease in V2a number and not only to a decrease in *Chx10* expression, as the other V2a marker Sox14 showed a similar reduction (Fig. 4p-v; *p* = 0.010). To assess whether this alteration could result from an expansion of the MN population at the expense of V2 INs, we quantified MNs in the constitutive mutant. However, the number of MNs was not increased in the absence of Lhx4 (Fig. 5l-o; *p* = 0.917). Consistently, the conditional loss of Lhx4 in MNs had no impact on V2a production (Fig. 4d-g; *p* = 0.76). Of note, no hybrid cells co-expressing MN and V2a markers [50, 51] were identified in any of these mutants (Figure S4). These results suggest that Lhx4 is necessary for proper differentiation of V2 INs and that its loss is not entirely compensated by the presence of Lhx3. To determine if Lhx4 was necessary in V2 precursors for the segregation between the MN and V2 IN lineages or was specifically acting in differentiating V2a INs, we quantified V2b INs in the same mutants. However, the loss of Lhx4, whether conditional or constitutive, had no impact on V2b IN production (Fig. 6a-o; *p* = 0.403, 0.195, 0.560, in order of appearance on the graph), indicating that Lhx4 is specifically acting in V2a IN differentiation. Consistently with our observations in the constitutive mutant (Fig. 5l-o), the conditional loss of Lhx4 in MNs or in V2 INs did not alter MN production (Fig. 5a-k; *p* = 0.736, 0.440, respectively), suggesting that Lhx3 is able to compensate for the loss of Lhx4 in this population. Taken together, these observations indicate that Lhx4 is critical for the differentiation of V2a INs and suggest that it is more efficient than its paralog Lhx3 in this process, whereas Lhx3 and Lhx4 can mutually compensate for the absence of the other during the segregation of the MN and V2 lineages [7, 8] and in differentiating MNs [4].

## Discussion

Paralog factors are proposed to promote robustness of biological processes by displaying redundant functionality when present in the same cells [1]. Here, we showed that Lhx3 and Lhx4 are both produced in differentiating MNs and in V2 INs during chicken or mouse spinal cord development. We demonstrated that Lhx4 and Isl2 can, as reported for Lhx3 and Isl1 [7, 8], form Lhx-NLI or Isl-Lhx-NLI complexes and can stimulate activation of the corresponding enhancers. However, Lhx4 seemed more potent than Lhx3 in stimulating the V2 TeRE enhancer. Furthermore, although Lhx4 and Lhx3 were both able to stimulate V2a IN differentiation in chicken, Lhx4 was more efficient than Lhx3 in this process. Consistently, the loss of Lhx4 in mouse embryos resulted in a reduced number of V2a INs while it had no effect on V2b or MN production. This suggests that the loss of Lhx4, while compensated by the presence of Lhx3 in precursors and in MNs, was not entirely compensated in V2a INs. Lhx4 is therefore necessary for a proper differentiation of V2a INs and likely surpasses Lhx3 in its ability to promote V2a IN fate in the developing spinal cord.

Lhx3 and Lhx4 constitute one of the pairs of LIM-HD transcription factors displaying high sequence conservation, with 77% and 86% identity shared by the first and second LIM domains respectively, and 95% identity by the homeodomain [20, 21], suggesting that they exert very similar functions. As numerous paralog factors, Lhx3 and Lhx4 are detected in the same cells in multiple developing or adult tissues [22, 26, 36]. In the mouse spinal cord, Lhx4 and Lhx3 have been initially co-detected in differentiating MNs and in ventral INs of an unidentified subtype [4]. These ventral cells were more recently characterized as newly-born V2 precursors and differentiating V2a INs [7, 52], and we confirmed that the distribution of Lhx4 in MNs and in V2 INs is conserved between mouse and chicken embryonic spinal cord. Lhx3 and Lhx4 have been shown to act redundantly in spinal MNs to regulate their localization and determine the ventral projections of motor axons [4]. Consistently, MN differentiation was not altered in the MN-specific *Lhx4* mutants nor when Lhx4 was absent from both MNs and V2a INs in *Lhx4* constitutive mutants. Furthermore, activation of the HxRE MN enhancer and stimulation of MN differentiation was similar between Lhx4- and Lhx3-containing complexes. Thus, Lhx3 and Lhx4 seems to exert identical functions in MN differentiation and the absence of Lhx4 is compensated by the presence of Lhx3.

**Fig. 7:**
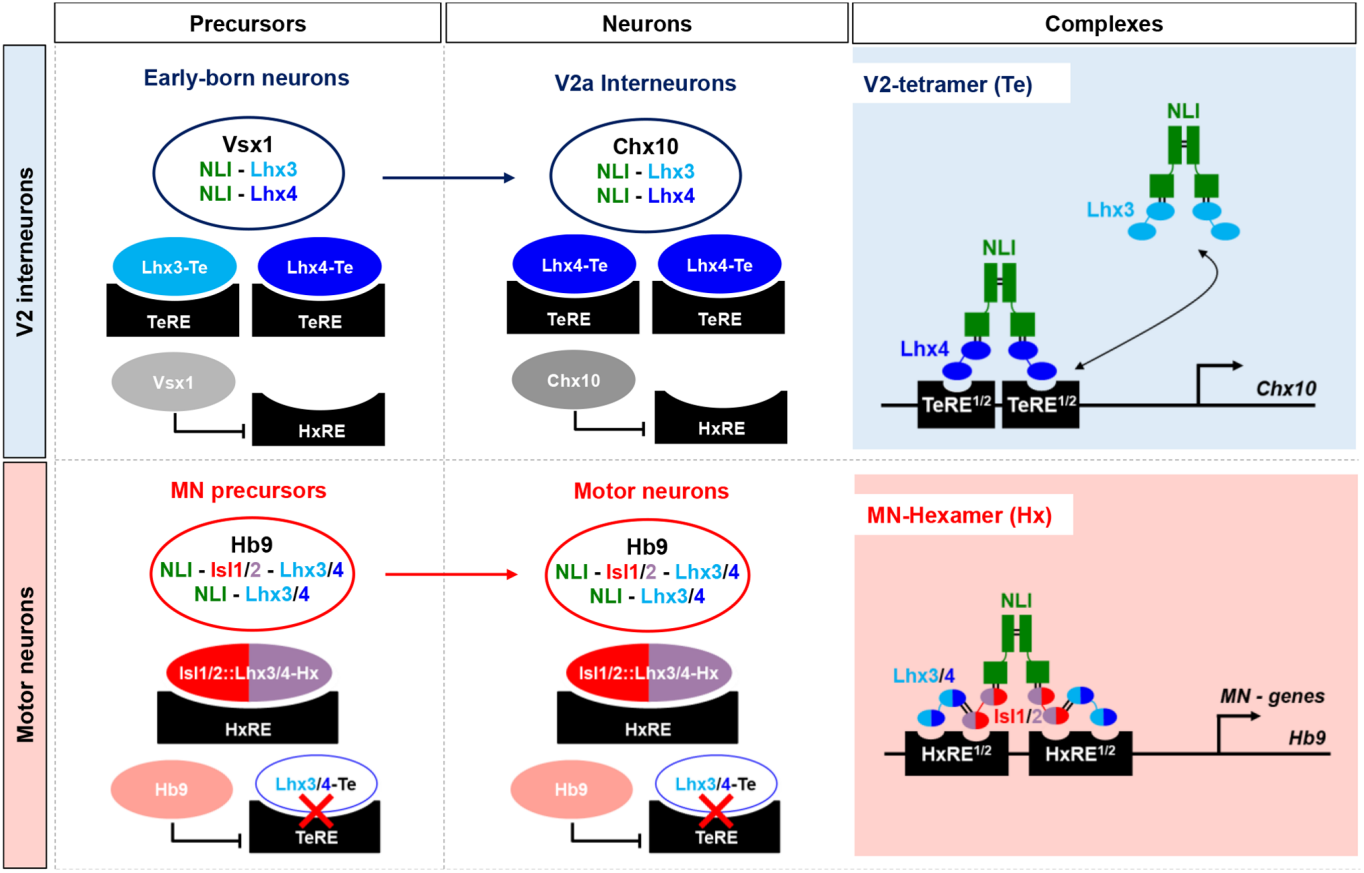
Working model for the contribution of Lhx factors to V2a IN and to MN differentiation. Schematic representation of V2 IN and MN identity specification and consolidation. In MN or V2 IN precursors, Isl1/2- and Lhx3/4-containing hexameric complexes promote MN fate, whereas Hb9 prevents activation of the TeRE enhancer and of the V2a differentiation program, and Lhx3/4-containing tetrameric complexes stimulate V2 differentiation while Vsx1 prevents illegitimate stimulation of the HxRE. During V2a differentiation, Lhx3- and Lhx4-containing tetrameric complexes promote V2a fate, whereas Chx10 prevents activation of the HxRE enhancer and of the MN differentiation program. Lhx4 is as efficient as Lhx3 in stimulating MN development but more potent than Lhx3 for stimulating V2a IN differentiation

However, Lhx3 and Lhx4 were also reported to exert distinct functions in different cell types during embryonic development [2, 3, 36, 37]. Consistently, the human syndromes resulting from *Lhx3* or *Lhx4* mutations are not identical, although partly similar [26]. Our observations support that Lhx4 is more efficient than Lhx3 in stimulating the activity of the TeRE V2 enhancer and promoting the differentiation of V2a INs, but not V2b INs, following overexpression in the chicken embryonic spinal cord. In addition, the absence of Lhx4 was not compensated by the presence of Lhx3 regarding V2a differentiation, whether in the V2-conditional or in the constitutive mutants. In contrast, the production of V2b INs remained unaffected in *Lhx4* gain-of-function or loss-of-function experiments. This suggests that Lhx4 does not exert early specific functions, different from those of Lhx3, in the consolidation of cardinal V2 IN versus MN identity, as its absence does not alter all the V2 populations, but could specifically stimulate V2a differentiation, more efficiently than Lhx3. These differences may result from differential binding abilities of these LIM-HD factors to their protein partners or to chromatin. Although they are both able to associate with Isl1 [2, 36], Lhx3 and Lhx4 show slight differences in their capacity to bind other LIM-HD factors [32, 33]. Isl1/2 can sequester the Lhx3/4 binding domain of NLI, preventing direct interaction between NLI and Lhx paralogs and promoting Isl-Lhx binding (Fig. 7), thereby favoring the aggregation of the hexameric complex in MNs [33]. Kinetic studies and modeling indicate that the complex formed by Isl2, which is critical for proper differentiation of different MN subsets [19, 30, 31, 34], and Lhx4 is eightfold more stable than its Isl1-Lhx3 equivalent [33], suggesting that Isl2-Lhx4 could constitute a more efficient complex to promote MN differentiation. However, our data rather suggest that the different combinations of Isl and Lhx factors are as efficient in activating the HxRE and in stimulating MN differentiation. Whether Lhx3 and Lhx4 have differential binding affinities for NLI in V2 tetrameric complexes or for protein interacting with these complexes remains to be determined. In addition, the relative formation of the different complexes is influenced by DNA binding. At equilibrium, in the absence of DNA, the formation of the 2Lhx3-2NLI tetrameric complexes prevails over that of hexameric complexes. Addition of DNA containing binding sites for the hexameric complex leads to an increased hexameric complex formation, which is reversed by subsequent addition of tetrameric complex binding sites [33]. This is in line with our observations that the 2Isl-2Lhx-2NLI hexameric complexes were more efficient in activating the HxRE and MN differentiation than the tetrameric complexes, independently from their composition (Figs. 1 and 3) [8, 10]. Whether the accessibility of the HxRE and TeRE to the corresponding binding complexes is different in MNs and in V2 INs, due to epigenetic modifications or preliminary binding of pioneer factors [53, 54], and impact the formation of the complexes in these two populations remains to be investigated.

Our results confirm that, although the hexameric complexes seem to constitute the major activators of the MN differentiation program, they can also activate TeRE (Fig. 1l) [8]. Illegitimate activation of the TeRE in MNs is prevented by the transcriptional repressor Hb9, which binds to TeRE and inhibits its stimulation by tetrameric complexes [8, 50, 51]. Symmetrically, V2 INs possess an original mechanism to ensure HxRE silencing throughout differentiation. In newly born V2 precursors, wherein Lhx3 and Lhx4 proteins are present, the Paired-Like CVC transcriptional repressor Vsx1 binds the HxRE and prevents its activation [16, 55]. When differentiation proceeds, V2 INs segregate into V2a or V2b populations upon the action of Notch signaling [56–60]. Lhx3 and Lhx4 are only maintained in V2a INs (Fig. 1) [7, 52]. In this population, the single paralog of Vsx1 in the mammalian genome, namely Chx10, takes over and prevents activation of the HxRE and of the MN differentiation program [9, 16]. Accordingly, V2b INs, wherein the expression of *Lhx3* and *Lhx4* is not maintained (Fig. 1) [7, 52], do not express Paired-like CVC repressors [55, 61]. Potential activators of the HxRE in V2 INs remain unknown, as hexameric complexes cannot form due to the absence of Isl proteins, although Pax6 and Nkx6.1, which are transiently maintained in V2 precursors, have been proposed [16].

Our observations additionally suggest that Lhx4 should be considered for in vitro differentiation protocols of spinal MNs or V2 INs for research or therapeutic purposes. The best protocols currently available to generate V2a INs in vitro enable the production of 25–45% of neurons with V2a characteristics [62, 63]. These methods involve either combinations of small-molecule agonists or antagonists of morphogen or signaling pathways [62], or a proneural Ngn-2-producing lentiviral vector combined with various pathway inhibitors [63]. These protocols may likely be improved by including factors that more specifically stimulate V2a IN differentiation, including Lhx3 [7, 8] or its more efficient paralog Lhx4 (the present work). Similarly, multiple differentiation protocols for spinal MNs involve Lhx3 in the cocktail of transcription factors used to promote MN phenotype (e.g [14, 64]). According to our present observations, these protocols could also be tested by replacing Lhx3 with Lhx4.

Finally, our observations underline the necessity to properly evaluate the contribution of each paralog factor to biological processes. Paralog factors that are co-expressed in the same cells are often *a priori* considered as having identical, and therefore possibly redundant, functions [1]. However, detailed investigations of their activities frequently unveil more complex or subtle situations wherein paralogs exert different functions or display significantly different activities in the same process [16, 65]. Hence, thorough investigation of the individual contribution of paralog factors to spinal cord development would provide a deeper understanding of the mechanisms involved and a more efficient transposition of this knowledge to other research fields, including in vitro differentiation of specific neuronal populations [14, 62–64] or in silico modeling of gene regulatory networks [66–68].

### Electronic supplementary material

Below is the link to the electronic supplementary material.

Supplementary Material 1

## Data Availability

The datasets generated during and/or analysed during the current study are available from the corresponding author on reasonable request.
