# Supplementary material for: Lhx4 surpasses its paralog Lhx3 in promoting the differentiation of spinal V2a interneurons
Source: Cell Mol Life Sci. 2024 Jul 6;81(1):286. doi: 10.1007/s00018-024-05316-x (PMC11335214; doi:10.1007/s00018-024-05316-x)
Supplement: Supplementary file 1 — Supplementary Material 1 [file 18_2024_5316_MOESM1_ESM.docx]

**Supplementary Information**

**Fig S1** Lhx4 and Lhx3 are co-detected with OC1 and OC2 in V2a subpopulations and in MNs. Immunofluorescence confocal pictures of mouse transverse embryonic spinal cord sections at stage E14.5. **a-b** OC1 is detected in V2a INs and in MNs containing Lhx3 or Lhx4. **a-d** OC2 is detected in V2a INs containing Lhx3 or Lhx4. **e-f** MafA is not detected in V2a INs producing Lhx3 or Lhx4 but is detected in MNs of the MMC containing the Lhx paralogs (arrows). Arrowheads indicate co-detection with Lhx4 or Lhx3 in V2a INs. Arrows indicate co-detection with Lhx4 or Lhx3 in the MMC. Scale bars = 50μm.

**Fig S2.1** Lhx4 can stimulate the differentiation of V2a/d INs more efficiently than Lhx3. Immunolabeling for V2a INs (Shox2+) on transverse spinal cord sections of chicken embryos electroporated with an empty vector or expression vectors for Lhx or Isl factors or the corresponding Isl::Lhx fusion proteins at stage HH15 and collected at stage HH26. Co-electroporation with pCMV-GFP is shown as an electroporation control. **a-c** As compared to an empty vector, Lhx3 and Lhx4 induce ectopic differentiation of V2a/d INs (Shox2+) (arrowheads) whereas Isl1, Isl2 or the different Isl::Lhx fusion proteins do not. Lhx4 is more efficient than Lhx3 to promote V2a/d differentiation. n≥3. + = electroporated side; - = control side; # = p<0.05; *** = p<0.001. Scale bars = 50μm

**Fig S2.2** Lhx4 can stimulate the differentiation of V2a INs more efficiently than Lhx3. Immunolabeling for V2a (Chx10+) or V2b INs (Gata3+) on transverse spinal cord sections of chicken embryos electroporated with an empty vector or expression vectors for Lhx or Isl factors or the corresponding Isl::Lhx fusion proteins at stage HH15 and collected at stage HH26. Co-electroporation with pCMV-GFP is shown as an electroporation control. **a-j** As compared to an empty vector, Lhx3 and Lhx4 induce ectopic differentiation of V2a INs (Chx10+) (arrowheads) whereas Isl1, Isl2 or the different Isl::Lhx fusion proteins do not. Lhx4 is more efficient than Lhx3 to promote V2a differentiation. **k-**t In contrast, Lhx and Isl factors or Isl::Lhx fusion proteins have no impact on V2b IN production (Gata3+). n≥5. + = electroporated side; - = control side; * or # = p<0.05; ## = p<0.01; *** or ### = p<0.001. Scale bars = 50μm

**Fig S3** In complexes with Isl1 or Isl2, Lhx4 can induce the differentiation of MNs as efficiently as Lhx3. Immunolabeling for MNs (Mnr2/Hb9+) on transverse spinal cord sections of chicken embryos electroporated with an empty vector or expression vectors for Lhx or Isl factors or the corresponding Isl::Lhx fusion proteins at stage HH15 and collected at stage HH26. Co-electroporation with pCMV-GFP is shown as an electroporation control. **a-j** As compared to an empty vector, all the Isl::Lhx fusion proteins can stimulate MN differentiation, as evidenced by the distribution of the newly-born MN marker Mnr2, whereas the Isl or Lhx factors alone do not. n≥3. + = electroporated side; - = control side; * = p<0.05; ** = p<0.01; *** = p<0.001. Scale bars = 50μm

**Fig S4** The loss of Lhx4 does not result in production of hybrid V2a/MN cells. **a-c** Immunolabeling of transverse spinal cord sections of MN-conditional Olig2|Lhx4^Δ/-^ mutant, V2-conditional Vsx1|Lhx4^Δ/-^ mutant, or Lhx4^-/-^ constitutive mutant embryos at E14.5. The loss of Lhx4 does not lead to production of hybrid cells co-expressing MN and V2a markers, respectively Isl1 and Chx10. Arrows indicate cells lacking co-detection of V2a and MN markers. Scale bars = 50μm.
